# KPC - 3 *Klebsiella pneumoniae* ST258 clone infection in postoperative abdominal surgery patients in an intensive care setting: analysis of a case series of 30 patients

**DOI:** 10.1186/1471-2253-13-13

**Published:** 2013-07-03

**Authors:** Paola Di Carlo, Gaspare Gulotta, Alessandra Casuccio, Gianni Pantuso, Maurizio Raineri, Clizia Airò Farulla, Sebastiano Bonventre, Giuliana Guadagnino, Daniela Ingrassia, Gianfranco Cocorullo, Caterina Mammina, Antonino Giarratano

**Affiliations:** 1 Department of Sciences for Health Promotion and Mother-Child Care “G. D’Alessandro”, University of Palermo, Via del Vespro 127, I- 90127, Palermo, Italy; 2 Department of General Surgery, Urgency, and Organ Transplantation, University of Palermo, Via del Vespro 127, I-90127, Palermo, Italy; 3 Department of Surgery and Oncology, University of Palermo, Via del Vespro 127, I-90127, Palermo, Italy; 4 Department of Experimental Biomedicine and Clinical Neuroscience, University of Palermo, Via del Vespro 127, I-90127, Palermo, Italy; 5 Intensive Care Unit, Department of Emergency, Critical Care and NeuroScience, University of Palermo, Via del Vespro 127, I-90127, Palermo, Italy

**Keywords:** *Klebsiella pneumoniae*, Carbapenemase, Abdominal surgery

## Abstract

**Background:**

Abdominal surgery carries significant morbidity and mortality, which is in turn associated with an enormous use of healthcare resources. We describe the clinical course of 30 Intensive Care Unit (ICU) patients who underwent abdominal surgery and showed severe infections caused by *Klebsiella pneumoniae* sequence type (ST) 258 producing *K. pneumoniae* carbapenemase (KPC-Kp). The aim was to evaluate risk factors for mortality and the impact of a combination therapy of colistin plus recommended regimen or higher dosage of tigecycline.

**Methods:**

A prospective assessment of severe monomicrobial KPC-Kp infections occurring after open abdominal surgery carried out from August 2011 to August 2012 in the same hospital by different surgical teams is presented. Clinical and surgical characteristics, microbiological and surveillance data, factors associated with mortality and treatment regimens were analyzed. A combination regimen of colistin with tigecycline was used. A high dose of tigecycline was administered according to intra-abdominal abscess severity and MICs for tigecycline.

**Results:**

The mean age of the patients was 56.6 ± 15 and their APACHE score on admission averaged 22.72. Twenty out of 30 patients came from the surgical emergency unit. Fifteen patients showed intra-abdominal abscess, eight anastomotic leakage, four surgical site infection (SSI) and three peritonitis. The overall crude ICU mortality rate was 40% (12 out of 30 patients). Twelve of the 30 patients were started on a combination treatment of high-dose tigecycline and intravenous colistin. A significantly lower mortality rate was observed among those patients compared to patients treated with approved dose of tigecycline plus colistin. No adverse events were reported with high doses of tigecycline.

**Conclusions:**

Critically-ill surgical patients are prone to severe post-surgical infectious complications caused by KPC-Kp. Timely microbiological diagnosis and optimizing antibiotic dosing regimens are essential to prevent worse outcomes. Further studies and well-controlled clinical trials are needed to define the optimal treatment of infections by KPC-Kp and, more generally, carbapenem-resistant bacteria.

## Background

Abdominal surgery carries significant morbidity and mortality, which is in turn associated with an enormous use of healthcare resources. Sepsis is a serious complication and may be diagnosed during pre-, intra-, or postoperative periods [1,2].

*Klebsiella pneumoniae* (Kp) is an emerging major pathogen in surgical settings, especially after emergency abdominal surgery [3,4].

In 2010, the first outbreak of *K. pneumoniae* carbapenemase (KPC)-Kp sequence type (ST)258 was reported in ICU patients in Palermo, Italy [5-7]. Since then, colonizations or infections with KPC-Kp have become endemic and have also been described in different healthcare settings, such as in surgical wards [8].

The aim of this study was to describe the clinical aspects of surgical KPC-Kp infections in patients who had undergone emergency or elective abdominal surgery. Risk factors for mortality and the impact of a combination therapy of colistin plus recommended regimen or higher dosage of tigecycline on the patients’ clinical course were evaluated.

## Methods

### Design and setting

This was a prospective case series study of post-surgical patients with monomicrobial bloodstream infections caused by KPC-Kp, admitted to the Intensive Care Unit (ICU) of the “Paolo Giaccone” University Hospital in Palermo, Italy.

The ICU under study is an 8-bed general ICU that provides care to emergency and elective surgery recipients, with approximately 250 patients admitted to the ICU annually.

In the period under study, the infection control policy in the ICU did not include routine surveillance cultures or screening of high-risk patients on admission. Special attention was given to hand hygiene measures, with an alcoholic hand rub solution placed in the proximity of every ICU bed or provided as a personal pocket dispenser. Furthermore, the ICU had a policy of infection control which included restricting the use of antibacterial drugs and clinical practice guidelines for infections with multidrug- resistant pathogens. A structured system for the surveillance of antimicrobial resistance has been implemented since June 2009.

A 3-monthly serial surveillance program for multidrug resistant Gram negative bacilli, including active surveillance cultures, has been carried out in the Surgical Emergency Unit since January 2010. High-risk patients are routinely screened on admission.

### Patients

We enrolled all postoperative abdominal surgery patients admitted from August 1, 2011 to August 31, 2012, who remained in the ICU for at least 48 hours and had at least two positive blood cultures for KPC-Kp*.* Organ failure was the leading cause of admission (65%) followed by monitoring/weaning from mechanical ventilation (35%).

All patients were treated with combined intravenous colistin (colistimethate sodium, 1 mg of colistin equals 12,500 IU) at a dosage of 5 mg/kg/day divided in three equal doses and tigecycline (recommended dosage regimen 100 mg initially, followed by 50 mg every 12 hours). The antimicrobial regimen was maintained or adapted according to the results of susceptibility testing. Patients who developed a severe intra-abdominal abscess were started on high-dose (initial dose of 200 mg then 100 q12) tigecycline combined with colistin. Because tigecycline MICs between 0.8-1 μg/ml are close to the upper limit of the European Committee on Antimicrobial Susceptibility Testing (EUCAST) susceptibility range, they were considered suboptimal and taken into account when making this decision (http://www.eucast.org) [9].

The study protocol was approved by the Ethics Committee of the Azienda Ospedaliero-Universitaria Policlinico “P. Giaccone”, Palermo, Italy. Before starting treatment, informed consent, also regarding publication of the patient’s details, was obtained from each patient’s next of kin in accordance with the principles of the Declaration of Helsinki.

### Data collection

General data, which included age, gender, infection site, date of admission and discharge from the surgery ward and ICU, chronic organ-function insufficiencies (as defined by the APACHE II scale), underlying conditions (i.e. alcoholism, smoking habit, diabetes mellitus, uncured malignancy and previous surgery), clinical outcome, microbiological findings, superinfection, reinfection and mortality were collected from ICU and surgical patient charts. Illness severity was measured with APACHE II scores based on the worst values in the first 24 h in the ICU [10]. Organ failure and severity of Multiple-Organ Dysfunction Syndrome (MODS) were evaluated using the Sequential Organ Failure Assessment (SOFA) scale on admission and during the subsequent clinical course.

Episodes of Ventilator Associated Pneumonia (VAP) in which KPC-Kp had been isolated were also included. The diagnosis of VAP was based on clinical and microbiological criteria [11]. A clinical suspicion of VAP was raised in patients with a Modified Clinical Pulmonary Infection Score (CPIS) >6 [11,12].

### Microbiological methods

Identification (ID) and antimicrobial susceptibility testing (AST) were routinely performed using a microdilution method (BD Phoenix™ Automated Microbiology System, Sparks, MD, US). Etest (bioMérieux) was used to determine susceptibility to colistin. Susceptibility and resistance categories were assigned following EUCAST guidelines.

Phenotypic confirmation of the presence of carbapenemases or overexpression of AmpC in combination with porin loss was obtained using a commercial synergy test (Rosco Diagnostica, Taastrup, Denmark). This test was carried out on putative carbapenemase-producing *K. pneumoniae* isolates based upon their screening cut-off values for meropenem, according to the recommended methods for detection of carbapenemases in Enterobacteriaceae [5-7,10]. ST258 was identified by Multi-locus Sequence Typing (MLST) [http://www.pasteur.fr].

### Statistical analysis

Frequency analysis was performed with chi-square test or Fisher’s exact tests, as needed. Univariate analysis of variance (ANOVA) was used for parametric variables. Risk ratios (RRs) and 95% confidence intervals (CIs) were calculated for associations with demographic and clinical variables. The continuous variables found to be independent predictors of mortality in the ICU were assessed using a linear regression model, and slope coefficients with their standard error were reported. Kaplan-Meier curve (log rank test) was plotted to estimate the cumulative incidence of mortality.

Results were expressed as mean ± standard deviation (SD) or median [interquartile range (IR)] for continuous variables or as percentages for categorical variables. Two-tailed tests were used and P values less than 0.05 were considered to be statistically significant. Data were analyzed using Epi Info software, version 3.2.2, (Centers for Disease Control and Prevention) and SPSS software (version 14.0; SPSS Inc., Chicago, IL, USA).

## Results

During the study period, we collected 30 cases of infection caused by KPC-Kp.

Patient characteristics are shown in Table 1. The mean age of the patients was 56.6 ± 15 years, and the APACHE score on admission averaged 23.4 ±1.7. Twenty of the 30 patients came from the surgical emergency unit. Fifty percent of these patients had been hospitalized in the previous two years. Four patients had chronic obstructive pulmonary disease (COPD) as underlying medical condition and two had diabetes mellitus. Smoking habit was present in the anamnesis of 16 patients, obesity in two, alcohol abuse in four and cocaine abuse in one patient. Multiple underlying conditions were observed in 6 patients.

**Table 1:** **Characteristics of 30 post-abdominal surgical ICU with infection by KPC-3 *Klebsiella pneumoniae *ST258 clone**

**Patients**	**Source of KPC-Kp positive isolate**	**Surgical infection**	**Underlying disease**	**Treatment**	**Outcome**
**(age yrs, gender)**					
01. 22, F	BAL^1^, Intraoperative Sample	SSI ^2^	Crush Syndrome	T + C^3^	Survived
02. 66, M	Intraoperative Sample	Intra-abdominal Abscess	Colon Cancer	HDT + C^4^	Survived
03. 54, F	Percutaneous Fluid	Pancreatic Abscess	Chronic Pancreatitis	HDT + C	Survived
04. 61, M	Intraoperative Sample	Intra-abdominal Abscess	Crohn’s Disease	HDT + C	Survived
05. 68, M	BAL, Intraoperative Sample	Intra-abdominal Abscess	Colon Cancer	HDT + C	Survived
06. 60, F	Percutaneous Fluid	Liver Abscess	Liver Cancer	HDT + C	Survived
07. 61, F	Intraoperative Sample	Intra-abdominal Abscess	Colon Cancer	HDT + C	Survived
08. 75, F	Drainage Fluid	Perianal Abscess	Rectal Cancer	HDT + C	Survived
09. 55, F	Intraoperative Sample	Intra-abdominal Abscess	Colon Cancer	HDT + C	Survived
10. 57, F	Drainage Fluid	Perineal Abscess	Rectal Cancer	HDT + C	Survived
11. 53, M	BAL, Intraoperative Sample	Intra-abdominal Abscess	Colon Cancer	T + C	Died
12. 67, F	Intraoperative Sample	Intra-abdominal Abscess	Colon Cancer	HDT + C	Survived
13. 56, M	Intraoperative Sample	Intra-abdominal Abscess	Crohn’s Disease	HDT + C	Survived
14. 45, M	BAL, Intraoperative Sample	Intra-abdominal Abscess	Pancreatic Cancer	HDT + C	Died
15. 57, F	Percutaneous Fluid	Pancreatic Abscess	Chronic Pancreatitis	T + C	Survived
16. 20, M	Percutaneous Fluid	Liver Abscess	Liver Cancer	T + C	Survived
17. 45, M	BAL, Wound Sample	SSI	Gastric By Pass	T + C	Died
18. 55, M	BAL, Intraoperative Sample	Peritonitis	Peritonitis	T + C	Died
19. 56, F	BAL, Abdominal Drain	Anastomotic Leak	Rectal Cancer	T + C	Survived
20. 65, F	BAL, Abdominal Drain	Anastomotic Leak	Rectal Cancer	T + C	Died
21. 84, M	BAL, Abdominal Drain	Anastomotic Leak	Rectal Cancer	T + C	Died
22. 29, M	Abdominal Drain	Anastomotic Leak	Colorectal Cancer	T + C	Died
23. 59, M	Intraoperative Sample	Anastomotic Leak	Crohn’s Disease	T + C	Survived
24. 61, M	BAL, Abdominal Drain	Anastomotic Leak	Colorectal Cancer	T + C	Died
25. 76, M	BAL, Intraoperative Sample	Peritonitis	Peritonitis	T + C	Died
26. 79, M	BAL, Abdominal Drain	Anastomotic Leak	Rectal Cancer	T + C	Died
27. 52, F	BAL, Wound Sample	SSI	Gastric By Pass	T + C	Died
28. 51, F	BAL, Wound Sample	SSI	Thyroid Cancer	T + C	Died
29. 52, F	Intraoperative Sample	Peritonitis	Peritonitis	T + C	Survived
30. 62, M	BAL, Wound Sample	Anastomotic Leak	Rectal Cancer	T + C	Survived

^1^**BAL**: Bronchoalveolar Lavage; ^2^**SSI**: Surgical Site Infection; ^3^ **T + C**: Tigecycline plus Colistin; ^4^**HDT + C**: High Dose Tigecycline plus Colistin.

**Figure 1:**
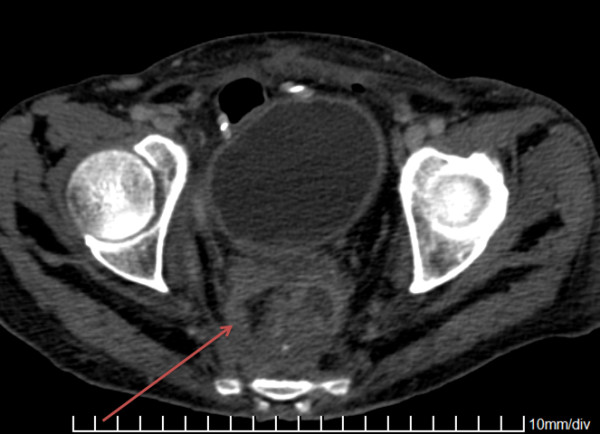
Computed Tomography scan of pelvis showed rectal abscess (arrowhead).

The most common surgical indication was rectal cancer in seven patients, followed by colon cancer in six patients. Other indications were less represented (Table 1). KPC-Kp post-surgical infections were as follows: intra-abdominal abscess in 15 cases, anastomotic leakage in eight, surgical site infection (SSI) in four and peritonitis in three patients, respectively. Figure 1 shows KPC-Kp infection in a patient with rectal cancer.

As shown in Table 1, KPC-Kp was isolated from all surgical samples as well as from broncho-alveolar lavage (BAL) in 50% of the patients who developed ventilator-associated pneumonia (VAP) during their ICU stay.

Fifteen of the 30 patients under study who had a history of previous hospitalizations proved to be colonized by KPC-Kp strains on admission to the surgical emergency unit. Four patients acquired KPC-Kp colonization during their stay in the surgical unit. Ten patients had also been exposed to carbapenems before admission.

All the KPC-Kp strains were resistant to imipenem (MIC ≥16 μg/ml), meropenem (MIC ≥32 μg/ml) and ertapenem (MIC ≥8 μg/ml). They were also resistant to amikacin (MIC ≥64 μg/ml), amoxicillin- clavulanic acid (MIC ≥32 μg/ml), cefepime (MIC ≥8 μg/ml), cefotaxime (MIC ≥8 μg/ml), ceftazidime (MIC ≥64 μg/ml), ciprofloxacin (MIC ≥4 μg/ml), levofloxacin (MIC ≥8 μg/ml), piperacillin-tazobactam (MIC ≥128 μg/ml), tobramycin (MIC ≥16 μg/ml), gentamicin (MIC ≥8 μg/ml) and trimethoprim-sulfamethoxazole (MIC ≥320 μg/ml). Before starting combined antibiotic treatment, all the strains isolated from blood samples were susceptible to colistin (MIC ≤0.5 μg/ml) and tigecycline (MIC ≤0.5 μg/ml). After starting the treatment, five strains of KPC-Kp showed tigecycline MICs of 0.8 – 1 μg/ml.

The average duration of treatment with a combination of tigecycline and intravenous colistin was 18 ± 6.5 days. Twelve patients were administered a combined treatment for more than 21 days. Two surviving patients were treated with prolonged courses of combined antibiotic treatment, with more than twenty-day intervals between courses. Twelve patients received a combination treatment of high-dose tigecycline and intravenous colistin. They included the five cases with KPC-Kp isolates exhibiting tigecycline MICs >0.5 μg/ml.

**Figure 2:**
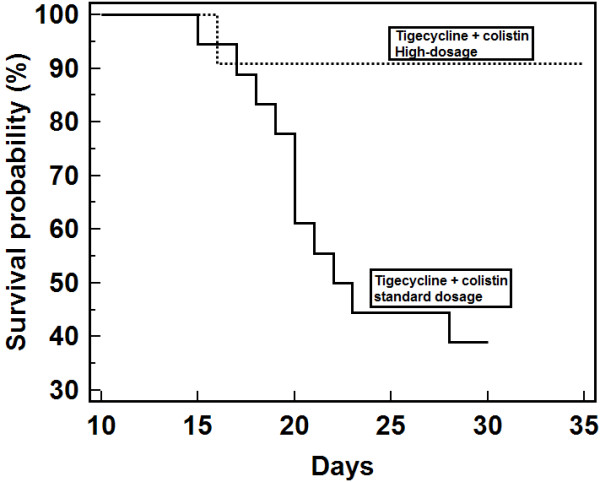
Kaplan-Meier survival curves show significantly lower mortality among patients treated with a combination therapy of high-dosage tigecycline plus colistin compared with those treated with recommended dosage of tigecycline plus colistin (log-rank test, p = 0.0035).

Overall crude ICU mortality rate was 40% (12 out of 30 patients). Risk factors for mortality in the ICU were assessed (Table 2). Using univariate analysis, a better outcome was associated with the presence of a surgical drainage, and a worse one with a higher APACHE II score and VAP. Treatment with high doses of tigecycline was associated with lower mortality (P = 0.005). Klapan-Meier curve showed that patients treated with high doses of tigecycline had a significant favorable outcome (log-rank test, p = 0.0035) (Figure 2).

**Table 2:** **Univariate analysis of risk factors associated to mortality in post-abdominal surgical ICU patients with infection by KPC-3 *Klebsiella pneumoniae *ST258 clone**

		**No. (%) of patients**			
**Variable**	**Dead (n = 12)**	**Survivors (n = 18)**	**Risk ratio**	**95% CI**	***P *value**
Male gender	8 (66.7)	7 (38.9)	1.57	0.84-2.92	0.080*
Age, years, mean (SD)	57.1 (17.7)	56.0 (13.9)	0.001	−0.011-0.14	0.895^
LOS in ICU, days, median (IQR)	20 (18.5-22.5)	18.5 (13–30)	0.005	−0.017-0.027	0.644^
LOS in surgery ward, median (IQR)	6.5 (3.5-8)	8 (5–10)	−0.051	−0.112-0.010	0.098^
Previous hospitalization	6 (50.0)	11 (61.1)	0.636	0.13-3.03	0.570*
Surgical drainage	2 (16.7)	12 (66.7)	0.44	0.22-0.85	0.005*
Underlying conditions					
Smoking	5 (41.7)	11 (61.1)	0.73	0.39-1.35	0.160*
Solid tumor	6 (50.0)	12 (66.7)	0.42	0.09-1.94	0.150*
APACHE II score, mean (SD)	24.5 (0.67)	22.7 (1.8)	0.147	0.051-0.242	**0.004^**
Infection type					
Septicemia	3 (25.0)	11 (61.1)			
VAP	4 (33.3)	6 (33.3)			
Septicemia + VAP	5 (41.7)	1 ( 5.6)	8.33	1.03-67.1	0.030*
Intestinal carriage of KPC-Kp	6 (50.0)	11 (61.1)	0.85	0.44-1.65	0.320*
Tigecycline + colistin					
High dosage	2 (16.7)	12 (66.7)	0.44	0.22-0.85	0.005*

Data are expressed as No. (%), unless otherwise defined;* logistic regression; ^ linear regression.

Abbreviations: *CI* confidence interval, *SD* standard deviation, *LOS* length of stay, *IQR* interquartile range, *COPD* chronic obstructive pulmonary disease, *APACHE* Acute Physiology and Chronic Health Evaluation, *VAP* ventilator associated pneumonia.

## Discussion

The results of our study draw attention to two important issues: the impact of KPC-Kp bloodstream infection in ICU patients who have had abdominal surgery, and the use of standard or high doses of tigecycline in combination with polymyxins to treat infection due to KPC-Kp in patients with serious surgical complications, such as intra-abdominal abscesses [1,2,7]. Moreover, our study highlights additional postoperative infectious complications, such as colorectal anastomotic leakage, which can be responsible for severe sepsis [13].

Some results appear to be relevant. Firstly, in all patients, sources other than blood samples yielded KPC-Kp strains and all clinical isolates proved to belong to the pandemic clone KPC-Kp ST258. This confirms our previous reports suggesting that this strain may have a possible selective advantage over other MDR pathogens in the hospital setting [5,6]. Moreover, about 50% of our surgical patients were colonized by KPC-Kp at admission and had a history of previous hospitalization. Ten patients had also received carbapenem therapy in the past. This is consistent with data from surveillance studies that report patients colonized with MDRGN before hospital admission [5,8] and with previous literature data describing the prolonged carriage of multidrug resistant bacterial strains following hospital discharge [8,14,15].

Secondly, half of our patients had monomicrobial intra-abdominal abscess due to KPC-Kp. As in our previous report [8], this observation is consistent with those of other authors, such as Hirsch [16], who describe even higher clinical success rates when using polymyxins in combination.

In our study, we report on the use of high-dose tigecycline in patients with intra-abdominal abscess, especially in those with a less favorable clinical course and with KPC-Kp isolates showing MICs of tigecycline near the upper limit of the EUCAST susceptibility range http://www.eucast.org). Since tigecycline is one of the few antibiotics which is generally active against KPC-Kp ST258 epidemic strain, optimal dosing is critical for maximal therapeutic effectiveness. There is still some debate over appropriate dosage and the most favorable pharmacokinetic/pharmacodynamic profiles in some kinds of infection, such as MDR *K. pneumoniae* /*Acinetobacter baumannii* urosepsis/bacteremia [17,18]. Some preliminary experiences with loading doses of 200 mg and 400 mg, then decreased by 50% to achieve a maintenance dose (100 mg or 200 mg every 24 hours respectively) seem to guarantee serum concentrations (3 and 6 mg/L respectively) and urine concentrations (0.6 and 1.2 mg/L respectively) that are two and four times higher than those attained with the usual doses [18].

Tigecycline has been approved for the treatment of intra-abdominal and skin and soft tissue infections (and for community-acquired pneumonia in the USA), although a warning was recently issued by the FDA (http://www.fda.gov) and the European Medicines Agency (EMA, http://www.ema.europa.eu), after clinical trials indicated a higher mortality rate in patients treated with tigecycline compared to control groups. A recent meta-analysis has confirmed increased mortality and has also indicated that, compared to other antibiotics, tigecycline has higher rates of clinical failure, septic shock, superinfections and other adverse events that can lead to therapy with the drug being discontinued. [19]. Yahav et al. advise against using tigecycline as monotherapy, especially in the treatment of serious infections [19,20].

Prolonged plasma half-life (24–48 hours) and pharmacodynamic index (AUC/MIC) suggest, however, that higher doses of tigecycline should be used. Nevertheless, the off-label use of tigecycline and higher doses should be reserved for the treatment of multidrug resistant Gram negative infections in patients with serious intra-abdominal infections, such as abscesses.

As we recently reported, we observed KPC-Kp isolates, which developed increasing tigecycline MICs after treatment [21]. It is therefore important to carefully evaluate the benefit of a higher dose of tigecycline or other treatment options in this complex cohort of patients.

Thirdly, our mortality rate was 40%, which is higher than reported by other studies [22]. Nonetheless, our patients were particularly complicated and showed at least one additional site of KPC-Kp infection, such as severe intra-abdominal infection. In 16 cases, a KPC-Kp VAP was also present. Although numerous studies have shown that VAP frequently complicates the ICU patient’s course, the issue of whether or not critically-ill patients are at an increased risk of death because of the acquired pneumonia remains controversial [23]. In a large study analysis, VAP-attributed mortality was higher in surgical patients and patients with intermediate (but not high) Simplified Acute Physiologic Score II values on ICU admission [24]. In our study, mean APACHE II score was higher in non-survivors than in survivors. It is postulated that both derangements in acute physiology and severity of underlying diseases at the time of admission could be responsible for mortality, rather than the underlying conditions. Despite improvements in our knowledge of the physiopathology of severe infection and the evolution of diagnostic methods, antibiotic therapy, postoperative care and surgical techniques, a substantial number of patients develop severe intra-abdominal infection and advanced stages of septic insult requiring admission to the ICU. The success of treatment of these conditions is multifactorial and the best antibiotic protocol may not suffice, unless adequate control of the focus of infection is achieved.

Fourthly, in our study underlying conditions, such as COPD, diabetes and lifestyle risk factors such as smoking, were often present. This is in line with the analysis of Huttenen [25], who reported a high prevalence of obesity and smoking and their association with poor prognosis in patients with bacteraemia.

Our study is not without limitations. It includes a small number of patients with very complex and heterogeneous conditions, as well as the bias associated with non-random enrollment, as the choice of treatment was based on the patients’ medical conditions.

Additionally, our results are not supported by studies comparing different protocols. Moreover, antibiotic therapy pattern and quality of spread of multidrug resistant pathogens in the ICU under study could not be generalized. A further limitation was that data on all patients with KPC-Kp bacteraemia admitted to our university hospital during the study period was not available.

Surveillance of healthcare associated infections has become an integral part of infection control and quality assurance of healthcare in many countries. Gastmeier *et al.* reported that effective surveillance could reduce the NI rate by about 20–30% on average [26]. Surveillance programs provide data about the organisms causing specific infections and their antibacterial drug resistance patterns. Moreover, such programs can guide clinical practices and infection prevention and control efforts in different geographic regions and clinical settings [6,26,27].

At present, hand hygiene and contact precautions have been improved, including dedicated patient-care equipment, disposable gloves and aprons, and environmental cleaning. The ICU has been thoroughly cleaned and respiratory equipment disinfected. Because of insufficient bed capacity, isolation is not feasible, but colonized or infected patients are spatially segregated. Compliance with hand hygiene and environmental cleaning procedures are monitored. Staff adherence is strongly promoted by weekly meetings between healthcare workers and the hospital infection control team.

## Conclusions

In summary, present-day surgical intensive care units continue to evolve with the latest technological advances. The modern ICU team provides continuous daily care to the critically-ill surgical patient in close cooperation with the surgical team. A vital role of the intensive care specialist is to establish and enforce protocols, guidelines and patient care pathways that ensure continued quality performance improvement. Despite the evolution of sophisticated adjuncts to healthcare and the improvement of structured critical care systems, the surgical critical care specialist continues to play a key role in improving patient outcomes.

## Abbreviations

MIC: Minimum inhibitory concentration; CI: Confidence interval; SD: Standard deviation; LOS: Length of stay; IQR: Interquartile range; COPD: Chronic obstructive pulmonary disease; APACHE: Acute Physiology and Chronic Health Evaluation; VAP: Ventilator associated pneumonia.

## Competing interests

The authors declared that they have no competing interests.

## Authors’ contributions

PDC designed the study and drafted the manuscript. GP and SM set up and implemented the study in the field and contributed to the interpretations of results. GG, GC and SB were in charge of the acquisition and interpretation of emergency surgical data; GP and CAF were in charge of the acquisition and interpretation of oncological surgical data. MR, DI and GG collected ICU data and MR was also in charge of the interpretation of intensive data. CM provided molecular typing data; AC supported epidemiological and statistical results. AG supervised the study and revised the manuscript. All authors have read and approved the final version of the manuscript.

## Pre-publication history

The pre-publication history for this paper can be accessed here:

http://www.biomedcentral.com/1471-2253/13/13/prepub
